# Phytoremediation of Explosives using Transgenic Plants

**DOI:** 10.4172/2157-7463.S4-001

**Published:** 2013-01-23

**Authors:** Santosh Kumar

**Affiliations:** Department of Pharmacology and Toxicology, School of Pharmacy, University of Missouri-Kansas City, USA

In the past century, several explosive compounds have become the major soil contaminants causing health and economic challenges to the society. In the U.S. alone millions of hectares of land are contaminated by various forms of explosives. The two most dangerous explosives, 2,4,6-trinitrotoluene (TNT) and hexahydro-1,3,5-trinitro-1,3,5-triazine (Royal Demolition Explosive; RDX), are major environmental pollutants in the USA and present distinct problems for bioremediation. The U.S. Environmental Protection Agency has classified these explosives as Class 1 carcinogens, which are retained in soil and are refractory to degradation. These explosives also seep into the underground water and may potentially contaminate drinking water. therefore, understanding the biology behind the metabolism of these explosives by microorganisms and plants is imperative to degrade these pollutants from the contaminated soil. Although the biotransformation of TNT has been studied in many organisms, particularly bacteria, the isolation of microorganisms that can solely utilize TNT for growth has remained elusive. In the recent past, several studies have identified and characterized plant enzymes involved in the reduction, conjugation and sequestration of TNT into plant cell walls [1]. In addition, transfer of bacterial nitro reductase genes to plants has shown significant promises to metabolize TNT into relatively less toxic compounds. The major goal of the scientific community is to identify enzymes that are capable of effciently degrading TNT and RDX, followed by the introduction of these enzymes in plants (transgenic plants) for effcient phytoremediation [2,3].

Phytoremediation using transgenic plants is a promising solution to many problems of environmental contamination [4,5]. It utilizes transgenic plants for uptake, sequestration, detoxification, or volatilization of inorganic and organic pollutants from soils, water, sediments, and possibly air. In nearly 20 years of research, many transgenic plants for phytoremediation have been produced, however, very few have shown promises in commercial applications [4,5]. The transgenic tobacco plant expressing bacterial pentaerythritoltetranitrate reductase is the first example of the dramatic increase in tolerance to explosives, glycerol trinitrate and TNT. In another example, transgenic tobacco and Arabidopsis plants expressing bacterial nitroreductases and cyanobacterial flavodoxin enzymes detoxify the explosives and other similar pollutant, 2,4-dinitrotoluene. However, these findings have not been yet translated to the field applicability for detoxification of these explosives. The most promising example of commercial application of transgenic plant for phytoremediation, thus far, is poplar transgenic plant for the degradation of TNT [3,6].

The recent results of the remediation of RDX has been obtained by the expression of bacterial xplA–xplB system in Arabidopsis, which showed the ability to remediate saturating levels of RDX from liquid culture and soil leachate [3,4,7]. is promising finding has facilitated the transfer of this technology into plants, such as poplar hybrids, which grows quickly and produces relatively high biomass. In addition, transgenic perennial grass species, which are fire resistant and are able to withstand the commotion by military equipment, may be used for the remediation of RDX. These grass species can also withstand relatively high TNT concentrations. Furthermore, recent research has demonstrated that transgenic aspen trees expressing a bacterial nitroreductase from *Pseudomonas putida* is capable of withstanding 2-fold higher level of TNT contamination compared to unmodified trees [4]. Since a variety of compounds secreted by plant roots support microbial diversity and growth, the introduction of transgenic plants into contaminated land will not only detoxify explosives, but also increase microbial diversity and population.

Although the research towards phytoremediation of explosives using transgenic plants is moving in the right direction, the scientific community encounters many problems, such as limited number of known explosives-metabolizing enzymes, relatively low activity of the natural enzymes, inability to introduce multiple genes simultaneously in plants, and limited number of plant species suitable for phytoremediation [8,9]. However, these problems also bring new opportunities for research to enhance the applicability of phytoremediation of explosives using transgenic plants [8–10]. These opportunities are: 1) technical advancement in gene sequencing, which have led to new developments in metagenomics, may find novel explosives-detoxifying enzymes with enhanced activities, 2) technical advancement in engineering of the natural explosive-degrading enzymes, such as cytochrome P450 with increased activities, using rational (site-directed mutagenesis) and irrational (directed evolution) approaches, may substantially increase the rate of degradation of explosives, 3) advancement in the cloning technology that introduce multiple explosive-degrading enzymes simultaneously into the plants, and 4) development in technology that can help create a variety of plant systems that are suitable for phytoremediation in diverse environments. Therefore, future research in these areas is needed to increase the applicability of transgenic plants in phytoremediation of explosives.
